# Optimisation of maintenance in delivery systems for cytostatic medicines

**DOI:** 10.1186/s12913-021-07093-w

**Published:** 2021-11-02

**Authors:** María Carmen Carnero, Andrés Gómez

**Affiliations:** 1 Technical School of Industrial Engineers, University of Castilla-la Mancha, Ciudad Real, Spain; 2 CEG-IST, Instituto Superior Técnico, Universidade de Lisboa, Lisbon, Portugal; 3 SERMAS, Madrid, Spain

**Keywords:** Maintenance strategy selection, Devices for the preparation of cytotoxic drugs, Multiple-criteria decision making, Fuzzy TOPSIS

## Abstract

**Background:**

The real-world application of maintenance in organisations brings together a number of maintenance policies in order to achieve the desired availability, efficiency and profitability. However, the literature mostly chooses a single maintenance policy, and so the decision process is not suited to the real conditions in the company to which it is applied. Our study takes a combination of maintenance policies as alternatives, and so conforms to the actual practice of maintenance in organisations. Furthermore, it introduces the possibility of including extra spare parts, or outsourcing maintenance policies. Although the selection of maintenance policies has been applied to many kinds of business and of machine, there is almost no instance of its application to hospitals, and it has never been applied to delivery systems for cytostatic drugs.

**Methods:**

The model uses the fuzzy Technique for Order of Preference by Similarity to Ideal Solution (TOPSIS), which is recognised as being highly suitable for solving group decision-making problems in a fuzzy environment. Fuzzy set theory is also considered to be more proficient than crisp numbers for handling the ambiguity, imprecisions, data scarcity, and uncertainty inherent in decisions made by human beings. The judgements required were obtained from a decision group comprising the heads of facilities maintenance, maintenance of medical equipment, health and safety at work, environment, and programming-admission. The group also included care staff; specifically, the heads of the main clinical services, and the medical supervisors. The model includes original criteria, such as Quality of health care, which measures impact on care as a function of mean availability of each alternative. It also considers Impact on hospital management via the criteria: Working environment in the organisation and Impact on health care; the former criterion measures equality among care services in the hospital, while the latter assesses the effect on regional health cover. The model was built using real data obtained from a state hospital in Spain. The model can also be easily applied to other national and international healthcare organisations, providing weights specific to the criteria. These are produced by a decision group from each healthcare organisation and the alternatives are updated in accordance with what is considered important in each hospital.

**Results:**

The results obtained from the model recommend changing the alternative that is currently in use, Corrective and Preventive Maintenance, to Corrective and Preventive Maintenance plus two spare hoods. This alternative would lead to an availability of 1 (the highest possible) in the systems for preparing personalised cytotoxic drugs, and so the quality of service is therefore very high. Additionally, it could offer services to all the users of the hospital, and also offer cover in the preparation of cytotoxic medicines to other hospitals in the catchment area.

**Conclusions:**

The results suggest the possibility that improvements to the support and logistical systems, which include maintenance, traditionally held to have no effect on quality of care, may be key to improving care quality, but also in reducing risk to patients, care and non-care staff, and the environment.

## Introduction

Anti-neoplastic, or cytotoxic, drugs are drugs which modify or inhibit the ability of cells to reproduce at one or another stage. This makes them useful for the treatment of malignant neoplastic diseases or cancer. They are, however, also used to treat non-cancerous diseases, such as psoriasis, multiple sclerosis, and systemic lupus erythematosus, which has led to increased use of these drugs [1]. Hospital pharmacy services are responsible, once a prescription has been written, for preparing the cytotoxic drugs, mixing, fortifying and diluting the original commercial product as required, to produce the personalised form suited to the needs of the patient. These products are then administered intravenously or orally (as chemotherapy).

Since the 80’s, the mutagenic, teratogenic and carcinogenic effects of cytotoxic drugs, if not handled and administered appropriately, have been well known [2]. These pharmaceutical products represent the most dangerous chemical risk factor in healthcare organisations, and are some of the most hazardous chemicals ever developed [1]. The preparation and delivery of cytotoxic drugs therefore carries a set of risks to the patient, the specialist care staff who prepare and administer them, and the non-care staff who perform cleaning and maintenance work on the areas/devices where these products are handled. For the patient, the risks arise from the narrow therapeutic margin of these drugs and their high toxicity, which leads to a high risk of harming the patient should there be any slight error. In the case of care and non-care staff, direct contact with these products could lead to them entering the bloodstream, with serious results. Hospital workers who handle them should therefore take strict safety measures to avoid any risk.

Given that the WHO, in its world report on cancer, predicts an increase in incidence of 50% by 2020, which would mean 15 million new cases [3], both patients and hospital staff in contact with these drugs have a significant increase in risk [4]. To these should be added the increasing number of professionals in contact with these products who are not directly involved in oncology, such as those who treat diseases in the fields of immunology, rheumatology, nephrology and dermatology, due to expanding use of these drugs in the treatment of non-malignant diseases [5].

For this reason, many associations, institutions and organisations have produced, and update regularly, manuals and lists of recommendations for handling cytotoxic drugs, (see for example [6, 7]). These look at questions of environmental protection, protection of healthcare or handling staff, and handling techniques [8]. The questions most frequently analysed, according to the literature review carried out by Bernabeu-Martínez et al. [9] are drug preparation, staff training and patient education and administration. However, most of the guidelines analysed consist only of recommendations on the handling of anti-neoplastics, and there is only one guide that addresses all the stages in the handling process of cytostatic drugs.

In 2016 the European Union published eleven recommendations aimed at reducing or preventing occupational exposure of staff to cytotoxic and other hazardous drugs [10]. It is the first document to put together a list of staff whose work may involve exposure to cytotoxic drugs. As well as care staff, this includes housekeeping staff, workers who ship, transport or receive hazardous drugs, and laundry and maintenance workers. There is, however, a serious failure to address questions related to the maintenance of delivery systems for cytotoxic drugs. Santillo et al. [11] produced the most recent guide to the handling of cytotoxic drugs in clinical areas: it includes recommendations for administering cytotoxic chemotherapy, Personal Protective Equipment (PPE), training of nursing staff, recommendations for infusions in infusion bags, recommendations for infusions in syringes, elastomeric infusors, sub-cutaneous injections, intrathecal injections, bladder installations, waste disposal, and cleaning of chemotherapy clinical areas, but there is no mention of which maintenance policy to apply. Only the Health and Safety Executive [12] of the United Kingdom has published in a manual the need for isolators to be adequately maintained by examination and testing at least every 14 months, as well as daily visual inspections. However, these stipulations are very generous, considering the consequences of a failure or fault in the systems.

Therefore, and despite its importance to patients, care and non-care staff and to the environment, there is no precedent in the literature studying maintenance policies in preparation systems for cytotoxic drugs. The maintenance of these systems can affect the availability of treatment, and thus, its effectiveness and patient recovery. They may be also be high risks to care staff and the environment should there be a failure of any component.

Maintenance, or asset management systems, may be defined as the combination of all technical and administrative actions, including supervisory action, intended to maintain an asset or return it to a fully functional state. Standard ISO 55000 states that it is the set of elements of the organisation that are interrelated with asset management, whose purpose is to establish an asset management policy, the aims of that policy, and the means by which these aims will be achieved [13].. A maintenance policy is a set of administrative, technical and managerial actions to be applied over the life-cycle of a device, used to guide maintenance-management decision making towards maintaining certain operating conditions of a machine, or intended to restore the machine to said conditions [14]. The choice of a maintenance policy is very important to availability, the quality of the service or product provided, the safety of the facilities and the staff, and the cost of facilities maintenance (which may be from 15 to 70% of production costs), efficiency, etc. [15]. However, despite these important implications, the literature on methods of selecting optimum maintenance strategies is limited [16].

Different types of maintenance policies can be defined:

- Corrective Maintenance (CM). Also known as breakdown maintenance or run-to-breakdown maintenance. It is carried out after a failure, with the aim of returning the machine to a state in which it can perform its desired function [17]. It is therefore characterised by passivity and a random dynamic; although there is no cost to detecting the state of the device, it leads to high costs in spares and maintenance activities, and high unavailability and lack of safety in facilities. Although organisations attempt to apply more efficient maintenance policies [16], there is still an underlying 10% of corrective maintenance that cannot be reduced.
- Preventive Maintenance (PM), implements preventive scheduled activities, a series of checks, replacements and/or component revisions with a frequency related to the failure rate, throughout the working life of the machine. This allows faults to be detected and repaired, or the faulty components to be repaired. However, this time-based maintenance involves high costs in spare parts and in specialised labour [16], which might, in some cases, be done without, as the machine shows no fault when the preventive activities are carried out.
- Condition Based Maintenance (CBM). This is based on the control of physical parameters (vibrations, temperature, water content of lubricant, etc.) of a working machine that can be recorded, periodically or continuously, by a set of sensors, to detect an abnormal situation, allowing maintenance resources to be prioritised and optimised. CBM minimises the cost in spare parts, reduces system downtime and increases system life span. However, it also requires a large initial investment in equipment (vibration analysers, sensors, ultrasonic testing devices, thermographic devices, etc.) and extra staff with extensive training in its use.
- Predictive Maintenance (PdM) predicts when to implement maintenance according to the monitoring data, which are analysed to find possible trends over time. This means that it is possible to predict when the controlled parameter value will reach or exceed the threshold values [15]. It combines appropriate scheduling of corrective maintenance with the state monitoring of condition based maintenance, and applies prognostics and health management (PHM) technologies to achieve better scheduled and highly cost-effective maintenance [16].
- Opportunistic Maintenance (OM), takes advantage of machine downtime to undertake maintenance activities, and carry out necessary checks and repair. This allows the frequency of stoppages to be reduced, by undertaking a series of repairs at the same time. It can also choose the most suitable time for the Production and Maintenance Departments to carry out the repairs. It is nevertheless true that its application requires a high level of coordination and backup from production staff.
- Total Productive Maintenance (TPM) is defined by Nakajima [18] as productive maintenance involving total participation, in addition to maximising equipment effectiveness and establishing a thorough system of planned preventive maintenance. Among these key points is getting the most efficient use out of the machine, involving the whole company in maintenance prevention, preventive maintenance and improvement-related maintenance, and encouraging the use of preventive maintenance based on small autonomous groups.
- Reliability Centred Maintenance (RCM). RCM is a systematic methodology for the allocation of efficient predictive and preventive maintenance aimed at preventing the dominant causes of failure of critical equipment. This can, in turn, lead to more acceptable levels of equipment availability and costs by reducing corrective maintenance [19].

The choice of maintenance policies is a complex decision, as it combines technical requirements for each machine with the company strategy and the established goals for availability, quality and safety. Thus, it is a decision which requires a great deal of thought, as different quantitative and qualitative criteria must be considered, which justifies the use of Multi-Criteria Decision Analysis techniques.

This article describes a model aimed at contributing to optimum choice of a combination of maintenance policies (both in-house and outsourced) and other actions for improvement, such as increasing the number of spares, in systems designed for personalised delivery of cytotoxic drugs. The model uses the fuzzy Technique for Order of Preference by Similarity to Ideal Solution (TOPSIS). The choice of this multicriteria technique over others is due to its adaptability to group decision-making processes [20, 21], as this study requires, because the judgements needed to apply fuzzy TOPSIS were obtained from a decision group comprising those in charge of a number of care and non-care departments. Fuzzy TOPSIS can be applied considering any number of criteria, which can be positive or negative, and qualitative or quantitative [22]; additionally, it includes the possibility of taking into account the uncertainty of such processes. The model was built from real data obtained from a state hospital in Spain.

The original aspects and contributions that should be highlighted in this research are the following:

1. The real application of maintenances policies consists of integrating several possible strategies to fulfil the requirements of the organisation in terms of availability, efficiency and profitability. However, it should be noted that in the literature, only Bertolini and Bevilacqua [23] and Ghosh and Roy [24] combine different maintenance strategies; The rest of the literature chooses a single maintenance policy, so the decision-making process is not adapted to the conditions which would allow real application in a company. This study considers as alternatives the combination of maintenance policies, so it is in line with the actual practice of maintenance in organisations.
2. The model is built using fuzzy TOPSIS. This method is highly suited to solving group decision-making problems in a fuzzy environment [20, 21]. The use of fuzzy set theory is recognised to be more proficient than crisp numbers for dealing with the ambiguity, imprecisions, data scarcity, and uncertainty inherent in decisions made by human beings [25]. This is because fuzzy set theory formalises the subjective and imprecise nature of human thinking by representing and quantifying vague information through a membership degree function.
3. Among the alternatives are options to outsource given maintenance policies and activities. This is common practice in many healthcare organisations, which outsource some maintenance services to the manufacturer or supplier of the medical equipment, and is not generally considered in the literature. This research considers the outsourcing of predictive maintenance by applying thermography analysis or hot spots in the systems analysed. This avoids having to purchase expensive thermography equipment and having to train personnel, but at the same time it guarantees the application of the most technological maintenance policy efficiently.
4. The selection of maintenance policies has been applied to very different types of companies and machines (see [13] or [26]), but usually a multicriteria model is used in manufacturing, and power and petrochemical plants. Its application to hospitals is almost non-existent and, in no case has it been applied to delivery systems for cytostatic drugs. There is no precedent for this in any study of maintenance, much less a model aimed at optimising decision making.
5. The model includes original criteria, such as Quality of health care, which measures the impact on care, as a function of mean availability of each alternative. It also considers the Impact on hospital management through the criteria: Working environment in the organisation and Impact on health care. While the first criterion measures equality between the care services of the hospital, the second evaluates the effect on regional health cover, if the service is classed as a reference in the regional health service, and the ability to attend to other areas assigned to the hospital. These questions have not been considered in any previous study.
6. The construction of the model used the judgements of a decision-making group made up of the main representatives of the different departments of the healthcare organisation: maintenance of facilities, maintenance of medical equipment, health and safety, environment, programming-admission, and care staff, consisting of clinical managers of central services and the supervisors of medical areas. This decision group provided the decision-making criteria, the descriptors used to evaluate the alternatives, the judgements necessary to obtain the weightings of said criteria, and the evaluations of the alternatives. The model thus incorporates the needs and characteristics required by the healthcare organisation to maintain a high level of quality of care, from a consensus of the entire hospital.
7. The model described can be easily applied to other healthcare organisations. It would be necessary to form a decision group from the organisation to give the judgements that will provide the weights of the criteria, as well as evaluating the alternatives in each criterion according to the particular conditions of the hospital maintenance service. Also, depending on what the decision group deems appropriate, they could include any additional criteria or modify any of those provided in this study.
8. The results obtained with the model propose the use of a combination of maintenance policies consisting of integrating Corrective and preventive maintenance plus the availability of two spare hoods. This alternative would provide an availability of 1 (the highest possible) in systems for the preparation of personalised cytotoxic drugs and the quality of service is therefore very high. Additionally, it could offer services to all the users of the hospital, and also offer cover for the preparation of cytotoxic medicines to other hospitals in the catchment area. Therefore, this shows how improvements to the support and logistical systems, which include maintenance, traditionally held to have no effect on quality of care, may be key not only to improving care quality, but also in reducing risk to patients, care and non-care staff, and the environment.

This paper is structured as follows. Firstly, there is a review of the literature on selection of maintenance policies. Next is a description of the fuzzy TOPSIS technique. Model for optimisation of maintenance in systems for preparation of cytotoxic drugs sets out the model; firstly, Markov chains are applied to systems for preparation of cytotoxic drugs at the hospital. Then fuzzy TOPSIS is applied, the criteria used and the matrices obtained are described. Results shows the results and the sensitivity analysis. Discussion shows the real implications of applying the results obtained with the model on quality of care at the hospital; finally come the conclusions and references.

## Background

In recent decades, maintenance has followed a trend with respect to sophistication and application cost similar to that of medical equipment. According to data from hundreds of hospitals, each hospital has an average of 15–20 new medical devices for each staffed bed, meaning an investment of some US$200–400,000/staffed bed, and the corresponding annual medical equipment maintenance cost is approximately 1% of the total hospital budget, which means that a 500-bed hospital spends on average around $5 million/year [27]. Thus, hospital maintenance has an important effect not only on costs, but also on the quality of care offered to patients.

The literature includes a large number of mathematical models (e.g. mathematical programming, genetic algorithms, etc.) for optimising maintenance [28], however, most of these are applied to a single maintenance policy, and they are also so difficult to solve that it is impossible to apply them in actual practice in organisations [29, 30]. Furthermore, the choice of maintenance policies should look, at the same time, at strategic, technical, economic and environmental aspects, as well as at safety and quality, etc. According to Almeida and Bohoris [31] and Martorell et al. [32], Multi-Criteria Decision Making (MCDM) techniques are well suited to this area of decision making, where there are many quantitative or qualitative criteria, or a combination of both, which also commonly clash with each other. MCDM techniques can support decision making in health care and contribute significantly to the transparency and consistency of decision making in the public sector [33], and it is also a flexible approach [34] as it can include multiple scenarios, stakeholders and decision makers.

According to the literature review of Adunlin et al. [35] on MCDM in health care, these techniques are used mainly in the diagnosis and treatment of diseases (39%), although Marsh et al. [36] show that it is used to support healthcare investment decisions, such as HTA, and national and local coverage decisions, up to 56% of contributions; it is also used, though less widely, in prescription decisions (22%), supporting authorisation (12%) and allocation of health research funding (2%). Additional applications MCDM in health care are related to formulary management, geographical information, pain management, performance measurement, priority setting, professional practice, public health and policy, resource planning, site selection, and supply chain. But the application of MCDM to maintenance in health care is not recognised. Only the review performed by Schmidt et al. [37], on the quality of research studies using Analytic Hierarchy Process (AHP) in health care, contains a contribution. A more recent literature review [38], does however recognise the existence of four contributions, out of a total of 66 analysed, which address equipment maintenance in healthcare organisations.

Table 1 shows the advantages and disadvantages of the application of different multicriteria crisp techniques. As can be seen, all multicriteria methods have advantages and disadvantages, so the selection of one method over another depends on each particular problem. Compared to the previous crisp multicriteria techniques, the use of fuzzy methods provides the possibility of including uncertainty due to incomplete, imprecise or vague information, ambiguities and inaccuracies inherent in the decision process, which otherwise would not be taken into account.

**Table 1:** Advantages and disadvantages of Multi-Criteria Decision Analysis techniques [39, 40]

Technique	Advantages	Disadvantages	Areas of application
Analytic Hierarchy Process (AHP)	Easy to use; scalable; the hierarchy structure can be easily adjusted to many sized problems; not data intensive; the use of pairwise comparisons allows more accurate information to be obtained from decision makers. Check inconsistency in the judgements	Problems due to interdependence between criteria and alternatives; can lead to inconsistencies between judgement and ranking criteria; rank reversal.	Performance problems, resource management, corporate policy and strategy, public policy, political strategy and planning.
Technique for Order of Preference by Similarity to Ideal Solution (TOPSIS)	A simple process; easy to use and programme; the number of steps remains the same regardless of the number of criteria; it returns stable results if input data are oscillating. More robust involving all stakeholders and interrelations between alternatives and objectives.	The use of Euclidean distances does not take into account the correlation of criteria; difficult to weight and to maintain consistency between judgements. Rank reversal.	Supply chain management and logistics, engineering, manufacturing systems, business and marketing, environment, human resources and water resources management.
Simple Additive Weighting (SAW)	Ability to compensate between criteria; intuitive for decision makers; simple calculation.	Estimates do not always reflect the real situations; the results obtained may not be logical.	Water management, business and financial management.
ELimination Et Choix Traduisant la REalité (ELECTRE)	Accounts for uncertainty and vagueness.	Its process and results may be complex to explain in layman’s terms; outranking can lead to the strengths and weaknesses of the alternatives not being directly identified. Rank reversal.	Energy, economics, environmental, water management and transportation problem.
Preference Ranking Organization METHod for Enrichment of Evaluations (PROMETHEE)	Easy to use; does not require assumption that criteria are proportionate.	Provides no procedure for obtaining weights for the criteria. Rank reversal.	Environmental hydrology, water management, business and finance, chemistry, logistics and transportation, manufacturing and assembly, energy, agriculture.
Measuring attractiveness through a categorical-based evaluation technique^[^(MACBETH)	Uses a value tree to structure a problem; the use of pairwise comparisons allows more accurate information to be obtained from decision makers. Avoids inconsistency in the judgements. Uses two reference levels for more objective judgements.	Its application is time-consuming.	Airport management, credit scoring, and strategic town planning, maintenance, energy, water management.
VIekriterijumsko KOmpromisno Rangiranje (VIKOR)	Able to establish the stability of decision performance; aggregate function is closest to the best solutions; the criteria selected may not allow homogeneous aggregation	Searching for the compromise ranking order; use of complex linear normalisation process; needs initial weights.	Renewable energy, service quality, earthquake sustainable reconstruction, material selection, land-use strategy

Fuzzy TOPSIS has been chosen over other fuzzy methods, most importantly fuzzy AHP, which is the technique applied to a greater extent in the fuzzy environment. TOPSIS makes full use of attribute information, it provides a cardinal ranking of alternatives and it does not require that the attribute preferences be independent [41], which fuzzy AHP does. Fuzzy TOPSIS does not impose any restriction on the number of criteria and alternatives [22]; however, Fuzzy AHP, like AHP, is limited to using nine criteria and alternatives so as not to compromise human judgement and its consistency. Although in this paper the model considers four alternatives, the aim is for it to be updated over time and, therefore, it is required that additional alternatives can be evaluated. TOPSIS logic is rational and understandable, the computation processes are simple, the concept allows the best alternatives for each criterion to be sought in a simple mathematical form, and the calculation of weights is incorporated into the comparison procedures [42]. Although there are many methods that allow the aggregation of judgments from more than one decision maker, aggregation of the weights of criteria and ratings of alternatives given by k decision makers is explicitly considered in the application procedure for fuzzy TOPSIS. Fuzzy TOPSIS is recognised as a suitable technique for solving group decision-making problems in a fuzzy environment [21]. Fuzzy TOPSIS is simple and yields an unarguable preference order, although each attribute should only have an increasing or decreasing utility [40]. Furthermore, it is the second most widely applied fuzzy MCDA technique in the literature, after fuzzy AHP, and it is recognised that this trend will continue [43]. It has been successfully applied to numerous real-world problems, as shown in the literature review by Salih et al. [44]. However, fuzzy TOPSIS has the problem of rank reversal. This consists of a change in the ordering among previously defined alternatives, after the addition or removal of an alternative from the previously ordered group. In order to solve this problem, García-Cascales and Lamata [42] propose absolute normalisation with the use of two fictional alternatives. One of them should contain 0 for all its values, and the other should have the maximum value in the decision matrix for all its values.

Comparing TOPSIS to other MCDM methods, for example PROMETHEE, the former was preferred, as the latter is time consuming and makes it difficult for decision makers to have a clear view of the problem, especially when many criteria are involved [45]. It also requires the weights of the criteria to be obtained by other methods, and it is necessary to choose the preference, the preference function, and the equivalence thresholds [46].

Most of the literature that uses MCDM in the choice of maintenance policies applies it to power plants for integrated gasification and combined cycle plants [47], a thermal power plant [28], the oil and gas industry [48, 49], the processing industry [50] or cogeneration systems [30]. It is applied to a lesser extent to weapons systems [51], centrifugal pumps operating in an oil refinery [52], in textile industry [53, 54], aircraft systems [55], a newspaper printing facility [56], the cement industry [57], a mining company [58], a paper manufacturing plant [59] or, for example, Hemmati et al. [60] who select the maintenance policy for an acid manufacturing company by means of a fuzzy Analytic Network Process (ANP) model or Asuquo et al. [61] who apply TOPSIS to marine and offshore machines. However, the characteristics of these industrial and business plants, and the equipment they use, are very different from the characteristics, machines and requirements of healthcare organisations, and so in many cases the criteria and techniques suggested cannot be used for hospital systems.

The precedents using MCDM techniques for the choice of maintenance policies in health care organisations are very few, and come from Taghipour et al. [62] who prioritise medical devices according to their criticality by applying AHP. Those devices with the lowest criticality are given low priority in a maintenance management programme. Those devices with high criticality are, on the other hand, more fully analysed, to determine suitable actions such as user training or equipment redesign. The criticality values obtained are used to establish guidelines for selecting appropriate maintenance strategies for different classes of device. The methodology was applied to 26 pieces of equipment with a variety of functions. Function, mission criticality, age, risk, recall and hazard alerts and maintenance requirements were used as criteria in the AHP model. Houria et al. [63] also prioritise medical equipment according to criticality. Degree of complexity of maintenance, function, risk, degree of importance of the mission, age, recalls and user errors and class of equipment were used as criteria in a fuzzy AHP model which allows the weight of each criterion to be obtained. Fuzzy TOPSIS is then applied to choose the most appropriate maintenance policy according to the closeness coefficient. Jamshidi et al. [64] apply fuzzy failure-mode and effect analysis to calculate a risk priority index. Next, an additive weighting model made up of seven criteria (age, usage-related hazards, utilisation, number of available identical devices, recalls and hazard alerts, function and maintenance requirements) was applied to obtain a TI for each medical device. AHP was used to obtain the weightings for the criteria. Finally, a maintenance planning diagram identifies the maintenance policy for each medical device. Mahfoud et al. [65] apply AHP Preference Ranking Organization METHod for Enrichment of Evaluations (PROMETHEE) to build a model to prioritise medical devices using multi-clinical expert judgements. Eight criteria were used: function, recalls and hazard alerts, utilisation, redundancy, age, technological obsolescence, maintenance requirements and a risk priority index. Carnero and Gómez [66] developed a multicriteria methodology for the combined choice of maintenance policies in dialysis subsystems associated with acute or chronic patients, infected with hepatitis B or C, using the Measuring Attractiveness by a Categorical Based Evaluation Technique (MACBETH) approach; the results show that the optimal decision is to apply corrective and preventive maintenance plus two reserve devices; the paper describes the implications of a change to the maintenance policies followed in the hospital for quality of care. Houria et al. [67] set out a model that integrates AHP and TOPSIS to classify the maintenance policies used in a hospital in Tunisia, and mixed integer problems MILP in order to select the most suitable maintenance policy for each medical device. Ighravwe and Oke [68] use stepwise weight assessment ratio analysis (SWARA), weighted additive sum product assessment (WASPAS), fuzzy axiomatic design (FAD), and the additive ratio assessment (ARAS) to choose the best maintenance strategy for public buildings (hospitals, research institutions, universities and polytechnics), and found as a result that the most suitable order is predictive maintenance, followed by preventive maintenance, condition-based maintenance and corrective maintenance. Previous contributions usually build models to be applied to different kinds of medical device, whose characteristics, use, redundancy, technological obsolescence or risks are varied. The study set out in this paper, however, uses a model designed exclusively for equipment used for the preparation of cytotoxic drugs, and incorporates specific information about failures, maintenance characteristics for mean repair times, etc. Questions such as the risk each type of equipment represents for patients, care staff, and other staff who might come into contact with it, and for the environment, were taken into account in the judgements produced by the experts at the hospital.

Furthermore, most of the previous studies chose the most satisfactory maintenance policy but did not consider that industry applies several policies simultaneously, and so it would be necessary to choose the best combination of policies, including the corrective policy, which should always exist, and the preventive, which is present in industry even when there are other more advanced policies, such as condition-based maintenance, reliability-centred maintenance or total productive maintenance.

## Methods

In 1965, Zadeh introduced the concept of Fuzzy Set (FS), as a class of objects with a continuum of membership degrees. This FS is characterised by a membership function that assigns to each object a degree of membership on the interval [0, 1] [69]. FS allows real-world decision problems to be formulated in which the alternative ratings and criteria weights cannot be precisely assigned due to the presence of unquantifiable, incomplete or unobtainable information, or partial ignorance [70]. Different types of FS have been introduced since 1965, some with the aim of solving the problem of constructing the membership degrees of the elements of the FS, and others aimed at representing uncertainty in a different way from that proposed by Zadeh [71]. Table 2 gives the definitions of the different types of FS explained in the literature. *X* is the domain, space or universe, *A*(*x*) the membership degree of the element *x* of the FS(A) and *μ*_*A*_(*x*) is a membership function. Atanassov intuitionistic fuzzy sets, vague sets, grey sets, interval-valued fuzzy sets, interval-valued Atanassov intuitionistic fuzzy sets, and some types of bipolar sets are particular cases of set-valued fuzzy sets. Additional properties and relations between types of fuzzy sets can be consulted in [71].

**Table 2:** Definition of different types of fuzzy set [71]

Acronym	Name	Definition
FS	Fuzzy Set	A FS (or type-1 fuzzy set) A on X is a mapping *A* : *X* → [0, 1], with *A*(*x*) the membership degree of the element x of the fuzzy set A. It can also be defined as *A* = {(*x*, *μ*_*A*_(*x*)) | *x* ∈ *X*} where *μ*_*A*_(*x*) : *X* → [0, 1].
T2FS	Type-2 Fuzzy Set	Is a FS for which the membership degrees are expressed as FS on [0, 1].
TnFS	Type-n Fuzzy Set	Is a FS whose membership values are type-(n-1) fuzzy sets.
AIFS	Atanassov Intuitionistic Fuzzy Set	An AIFS A on X is a mapping *A* : *X* → *D*([0, 1]) = {(*x*, *y*) ∈ [0, 1]^2^ | *x* + *y* ≤ 1}. *A*(*x*) = (*μ*_*A*_(*x*), *ν*_*A*_(*x*)) for all *x* ∈ *X*. *μ*_*A*_(*x*) is the membership degree of the element x to A and *ν*_*A*_(*x*) is the non-membership degree. Both values should satisfy the constraint 0 ≤ *μ*_*A*_(*x*) + *ν*_*A*_(*x*) ≤ 1.
BVFSL	Bipolar-Valued Fuzzy Set of Lee	A BVFSL on X is a mapping *A* : *X* → [−1, 1]. A bipolar scale is considered, with negative values held to be opposite to positive ones.
BVFSZ	Bipolar-Valued Fuzzy Set of Zhang	A BVFSZ on X is a mapping *A* : *X* → [0, 1] × [−1, 0]. *A*(*x*) = (*ρ*^+^(*x*), *ρ*^−^(*x*)) where *ρ*^+^: *X* → [0, 1], *ρ*^−^ : *X* → [−1, 0] and *ρ*^+^(*x*) + *ρ*^−^(*x*) ∈ [−1, 1]. BVFSZ involves two poles on two different scales. BVFSL is a particular case of BVFSZ
CFS	Complex Fuzzy Set	A on the universe X is a mapping *A* : *X* → *D* where *D* = {*re*^*is*^ | *r*, *s* ∈ [0, 1] *and i* = √ − 1}. That is, the membership function of a CFS A takes its values from the unit disk on the complex plane. Therefore, FS is a particular case of CFS.
FRS	Fuzzy Rough Set	In a universe X, and if R is a fuzzy similarity relation on X, let *A* ∈ *FS*(*X*). A fuzzy rough set on X is a pair (*R* ↓ *A*, *R* ↑ *A*) ∈ *FS*(*X*) × *FS*(*X*) where: • *R* ↓ *A* : *X* → [0, 1] *is given by R* ↓ *A*(*x*) = *inf*_*u* ∈ *X*_ *max* (1 − *R*(*u*, *x*), *A*(*u*)). • *R* ↑ *A* : *X* → [0, 1] *is given by R* ↑ *A*(*x*) = *sup*_*u* ∈ *X*_ *min* (*R*(*u*, *x*), *A*(*u*)).
FSS	Fuzzy Soft Set	A pair (F, A) is a FSS over X, where F is a mapping given by *F* : *A* → *FS*(*X*), where FS(X) denotes the set of all fuzzy subsets of X and A is a set of parameters.
GS	Grey Set	Defined in the same way as IVFS
HFS	Hesitant Fuzzy Set	This is a function that when applied to X returns a subset of [0, 1].
THFS	Typical Hesitant Fuzzy Set	This is a HFS where the membership degree of each of the elements is given by a finite and non-empty subset of [0, 1].
IT2FS	Interval Type-2 Fuzzy Set	When all *μ*_*A*_(*x*, *u*) = 1, then A is an IT2FS corresponding to *A*(*x*) = {(*u*, 1)| *u* ∈ *Jx* ⊆ [0, 1]} for every *x* ∈ *X*.
IVAIFS	Interval-Valued Atanassov Intuitionistic Fuzzy Set	If A on X is a mapping $A:X\to LL\left(\left[0,1\right]\right)=\left\{\left(\left[\underset{\_}{\mu },\overline{\mu}\right],\left[\underset{\_}{\nu },\overline{\nu},\Big\}\right]\right)|\left[\underset{\_}{\mu },\overline{\mu}\right],\left[\underset{\_}{\nu },\overline{\nu},\right]\in L\left(\left[0,1\right]\right)\ such\ that\ \overline{\mu}+\overline{\nu}\le 1\right\}$. Where *L*([0, 1]) denotes the set of all closed subintervals of the unit interval.
IVFS	Interval-Valued Fuzzy Set	If *L*([0, 1]) is the set of all closed subintervals of [0, 1], i.e., $L\left(\left[0,1\right]\right)=\left\{\left[\underset{\_}{x},\overline{x}\right]|\left(\underset{\_}{x},\overline{x}\right)\in {\left[0,1\right]}^2\ \mathrm{and}\ \underset{\_}{x}\le \overline{x}\right\}$. An IVFS A on X is a mapping *A* : *X* → *L*([0, 1]).
mPVFS	m-Polar-Valued Fuzzy Set	If m ≥ 2. An mPVFS on X is a mapping *A* : *X* → {1, …, *m*} × [0, 1]
NS	Neutrosophic Set	A NS A on X is a mapping *A* : *X* → [0, 1]^2^.
PFS	Pythagorean Fuzzy Set	A PFS A on X is a mapping *A* : *X* → {(*x*, *y*) ∈ [0, 1]^2^ | *x*_2_ + *y*_2_ ≤ 1}.
SVFS	Set-Valued Fuzzy Set	This is a FS for which membership degrees are expressed as subsets on [0, 1]. It can also be defined as A on X as a mapping *A* : *X* → 2^[0, 1]^\{∅}, where 2^[0, 1]^ is the power set of [0, 1], that is, the set of all subsets of [0, 1].
SS	Shadow Set	Given *A* ∈ *FS*(*X*), a shadow set B induced by A is an IVFS on X such that the membership degree of an element *x* ∈ *X* is either [0, 0], [1, 1] or [0, 1];
VS	Vague Set	Introduced by Gau and Buehrer in 1993. In 1994, it was shown to be the same as AIFS.

A fuzzy number is a normal, convex membership function. That is, its membership function is piecewise continuous and there exists at least one ${x}_0\in \mathfrak{R}$ satisfying ${\mu}_{\overset{\sim }{A}}\left({x}_0\right)=1$, where A is a fuzzy set [72]. A fuzzy number is a quantity whose values are imprecise. The literature includes a variety of fuzzy numbers, with their membership functions, such as triangular fuzzy numbers, trapezoidal fuzzy numbers, pentagonal fuzzy number, heptagonal fuzzy numbers, diamond fuzzy number, and pyramids. In this study, Triangular Fuzzy Numbers (TFN) are used due to their relative simplicity of calculation [73] and their wide application in representing the opinions of decision makers. A TFN $\overset{\sim }{A}$ can be defined as a triplet (*l*, *m*, *u*) with a membership function ${\mu}_{\overset{\sim }{A}}(x):\mathfrak{R}\to \left[0,1\right]$ as shown in Eq. (1) [74]. With *l* ≤ *m* ≤ *u,* where *l* and *u* are the lower and upper values of fuzzy number $\overset{\sim }{A,}$ and m is the modal value.

1 $$ {\mu}_{\overset{\sim }{A}}(x)=\left\{\begin{array}{c}0,\\ {}\left(x-l\right)/\left(m-l\right),\\ {}\left(x-u\right)/\left(m-u\right),\\ {}0,\end{array}\right.{\displaystyle \begin{array}{c}\ \\ {}\ \\ {}\ \\ {}\begin{array}{c}\ \\ {}\ \\ {}\ \end{array}\end{array}}{\displaystyle \begin{array}{c}x<l\\ {}l\le x\le m\\ {}\ m\le x\le u\\ {}x>u\end{array}} $$

If $\overset{\sim }{A}=\left({l}_1,{m}_1,{u}_1\right)$ and $\overset{\sim }{B}=\left({l}_2,{m}_2,{u}_2\right)$ are considered to be two TFNs, then the operational laws of these triangular fuzzy numbers are as follows [75]:

2 $$ \overset{\sim }{A}\oplus \overset{\sim }{B}=\left({l}_1+{l}_2,{m}_1+{m}_2,{u}_1+{u}_2\right), $$

3 $$ \overset{\sim }{A}\ominus \overset{\sim }{B}=\left({l}_1-{u}_2,{m}_1-{m}_2,{u}_1-{l}_2\right), $$

4 $$ \overset{\sim }{A}\otimes \overset{\sim }{B}\approx \left({l}_1{l}_2,{m}_1{m}_2,{u}_1{u}_2\right), $$

5 $$ \overset{\sim }{A}\oslash \overset{\sim }{B}\approx \left({l}_1/{u}_2,{m}_1/{m}_2,{u}_1/{l}_2\right), $$

6 $$ {\overset{\sim }{A}}^{-1}\approx \left(1/{u}_1,1/{m}_1,1/{l}_1\right)\  for\ l,m,u>0, $$

7 $$ k\otimes \overset{\sim }{A}\approx \left({kl}_1,{km}_1,{ku}_1\right),k>0,k\in R, $$

Fuzzy TOPSIS was proposed by Chen [76] from a modification to the diffuse medium of the technique developed by Hwang and Yoon [77]. Fuzzy TOPSIS has been successfully applied in many real cases [61], such as to support outsourcing of logistics services [78], to choose locations for shopping malls in Turkey [79], or the relief gate for a dam in Greece [80], to assess health vulnerability caused by climate and air pollution in South Korea [81], to choose the best equipment maintenance service supplier [82], to assess the performance of human resources in technology and science in Asian countries [83], to carry out safety risk assessment procedures in sustainable engineering projects [84], to measure environmental conflicts in mining in Vietnam [85], to facilitate the selection of services in the cloud [86], etc.

The advantages of using fuzzy TOPSIS include the following ([61, 78]):

- It is an easy technique to understand.
- It is a realistic compensatory model which can include or exclude alternatives based on hard cut-offs.
- It is easy to include more criteria or functionality, with no need to start from scratch.
- The mathematical notions behind fuzzy TOPSIS are simple.

In a decision problem with criteria (*C*_1_, *C*_2_, …, *C*_*n*_) and alternatives (*A*_1_, *A*_2_, …, *A*_*m*_), the best alternative in fuzzy TOPSIS is that which has the shortest distance to a fuzzy positive ideal solution (FPIS) and is at the greatest distance from a fuzzy negative ideal solution (FNIS). The FPIS is calculated using the best performance values for each criterion and the FNIS looks at the worst performance values.

In fuzzy TOPSIS the criteria must be monotonic, so it should satisfy one of these two conditions [61]:

- As the value of the variable increases, so do those of other variables (similar to the benefit type criterion).
- As the value of the variable increases, the other variables decrease (similar to cost type criterion).

In fuzzy TOPSIS the decision makers use linguistic variables to obtain the weightings of the criteria and the ratings of the alternatives. If there is a decision group made up of *k* individuals, the fuzzy weight and rating of the *k*th decision maker with respect to the *it*h alternative in the *j*th criterion are respectively:

8 $$ \tilde{w}_{j}^k=\left({w}_{j1}^k,{w}_{j2}^k,{w}_{j3}^k\right) $$

9 $$ \tilde{x}_{ij}^k=\left({a}_{ij}^k,{b}_{ij}^k,{c}_{ij}^k\right) $$

Where *i* = 1, 2, …, *m* and *j* = 1, 2, …, *n*.

The aggregate fuzzy weights $\tilde{w}_{ij}$ of each criterion given by *k* decision makers are calculated using Eq. (10).

10 $$ \tilde{w}_{j}=\frac{1}{K}\otimes \left(\tilde{w}_{j}^1\oplus \tilde{w}_{j}^2\oplus \dots \oplus \tilde{w}_{j}^k\right) $$

While Eq. (11) is used to calculate the aggregate ratings of the alternatives [87].

11 $$ \tilde{x}_{ij}=\frac{1}{K}\otimes \left(\tilde{x}_{ij}^1\oplus \tilde{x}_{ij}^2\oplus \dots \oplus \tilde{x}_{ij}^k\right) $$

Equation (12) shows a fuzzy multicriteria group decision-making problem which can be expressed in matrix format [76].

$$ D=\left[\begin{array}{cccc}{\overset{\sim }{x}}_{11}& {\overset{\sim }{x}}_{12}& \dots & {\overset{\sim }{x}}_{1n}\\ {}{\overset{\sim }{x}}_{21}& {\overset{\sim }{x}}_{22}& \dots & {\overset{\sim }{x}}_{21}\\ {}.& .& .& .\\ {}.& .& .& .\\ {}.& .& .& .\\ {}\tilde{x}_{m1}& \tilde{x}_{m2}& \dots & \tilde{x}_{mn}\end{array}\right] $$

12 $$ \overset{\sim }{W}=\left({\overset{\sim }{w}}_1,{\overset{\sim }{w}}_2,\dots, \tilde{w}_{n}\right) $$

Where $\tilde{w}_{j}$ and $\tilde{x}_{ij}$ are linguistic variables which can be described by triangular fuzzy numbers.

The weightings of the criteria can be calculated by assigning directly the linguistic variables shown in Table 3. The ratings of the alternatives are found using the linguistic variables of Table 4 [76].

**Table 3:** Linguistic variables for the weights [76]

Linguistic variables for the weights	Fuzzy number
Very Low (VL)	(0, 0, 0.1)
Low (L)	(0, 0.1, 0.3)
Medium Low (ML)	(0.1, 0.3, 0.5)
Medium (M)	(0.3, 0.5, 0.7)
Medium High (MH)	(0.5, 0.7, 0.9)
High (H)	(0.7, 0.9, 1.0)
Very High (VH)	(0.9, 1.0, 1.0)

**Table 4:** Linguistic variables for the ratings [76]

Linguistic variables for the ratings	Fuzzy number
Very Poor (VP)	(0, 0, 1)
Poor (P)	(0, 1, 3)
Medium Poor (MP)	(1, 3, 5)
Fair (F)	(3, 5, 7)
Medium Good (MG)	(5, 7, 9)
Good (G)	(7, 9, 10)
Very Good (VG)	(9, 10, 10)

The linear scale transformation is used to transform the various criterion scales into a comparable scale. And thus we obtain the normalised fuzzy decision matrix *R* [88].

13 $$ \overset{\sim }{R}={\left[\tilde{r}_{ij}\right]}_{mxn}\ i=1,2,\dots, m;j=1,2,\dots, n. $$

where

$\tilde{r}_{ij}=\left(\frac{l_{ij}}{u_j^{+}},\frac{m_{ij}}{u_j^{+}},\frac{u_{ij}}{u_j^{+}}\right) and\ {u}_j^{+}={\mathit{\max}}_i{u}_{ij}$ in the case of benefit type criteria.

$\tilde{r}_{ij}=\left(\frac{l_j^{-}}{u_{ij}},\frac{l_j^{-}}{m_{ij}},\frac{l_j^{-}}{l_{ij}}\right) and\ {l}_j^{-}={\mathit{\max}}_i{l}_{ij}$ in the case of cost type criteria.

Next, the weighted normalised decision matrix, $\overset{\sim }{V}$ is calculated, by multiplying the weightings of the criteria $\tilde{w}_{j}$, by the elements $\tilde{r}_{ij}$ of the normalised fuzzy decision matrix.

14 $$ \overset{\sim }{V}={\left[\tilde{v}_{ij}\right]}_{mxn}\ \mathrm{donde}\ \tilde{v}_{ij}=\tilde{x}_{ij}\otimes \tilde{w}_{j} $$

The distances ${d}_i^{+}$ and ${d}_i^{-}$ of each weighted alternative from the FPIS and FNIS are calculated using Eqs. (15) and (16).

$$ {d}_i^{+}=\sum \limits_{j=1}^n{d}_{\nu}\left({\overset{\sim }{\nu}}_{ij},{\nu}_{ij}^{+}\ \right)\ (15) $$

$$ {d}_i^{-}=\sum \limits_{j=1}^n{d}_{\nu}\left({\overset{\sim }{\nu}}_{ij},{\nu}_{ij}^{-}\ \right)\ (16) $$

where ${d}_{\nu}\left(\overset{\sim }{a},\overset{\sim }{b}\right)$ is the distance measured between the fuzzy numbers $\overset{\sim }{a}$ and $\overset{\sim }{b}$. This distance is calculated from Eq. (17) [88].

17 $$ {d}_{\nu}\left(\overset{\sim }{a},\overset{\sim }{b}\right)=\sqrt{\frac{1}{3}\left[{\left({l}_a-{l}_b\right)}^2+{\left({m}_a-{m}_b\right)}^2+{\left({u}_a-{u}_b\right)}^2\right]} $$

Finally, the closeness coefficient, *CC*_*i*_, is calculated for each alternative *i* using Eq. (18). This parameter allows the degree of fuzzy satisfaction to be evaluated for each Healthcare Organisation.

18 $$ {CC}_i=\frac{d_i^{-}}{d_i^{-}+{d}_i^{+}} $$

## Model for optimisation of maintenance in systems for preparation of cytotoxic drugs

The preparation systems for cytotoxic drugs analysed in this study are from a state-run hospital opened in 2005. The built area is 100,000 m^2^ making it one of the largest in Spain. It has 540 beds, 12 operating theatres, 24 intensive care beds, 4 paediatric intensive care units, 24 neonatal posts, 16 reanimation points, a dialysis unit, a major surgery out-patient unit with 23 stations, a haemodynamic and pacemaker insertion unit, a digital image treatment area, laboratories, a pharmacy, sterilisation unit, blood bank, 119 doctors’ surgeries, heliport, etc. It has a direct catchment area of 42 population centres, and offers health services directly to 174,550 inhabitants, and another 370,000 may be referred there from other hospitals. It is a reference in the region for eating disorders, nuclear medicine and its blood bank.

The multicriteria model requires information about the mean availability of the alternatives considered. Firstly, there is a description of the study with continuous time Markov chains carried out on the preparation systems for cytotoxic drugs at the hospital. Then, there is a description of the multicriteria model developed by fuzzy TOPSIS.

### Markov chains for the preparation systems for cytostatic drugs

The pharmacy is part of the care support services at the hospital, making medicines and cytostatics, and maintaining a general stock. The pharmacy includes systems for preparing cytotoxic drugs, and for storage and conservation of the vertical and horizontal kardex and the climate systems for pharmacotechnics and the electricity supply systems.

System 1 is used to prepare personalised medicines for oncological and other treatments, while System 2 prepares nutrients for the daily supply of tube-fed patients.

These systems are considered important to the hospital, as their working directly affects patients, care staff responsible for making medicines, the environment, and non-care staff carrying out maintenance, cleaning, refuse collection, etc.

Markov chains are widely used in research, as they allow systems to be modelled and their reliability, maintainability, availability and safety parameters to be estimated. This paper applies standard CEI IEC 61165 [89].

A continuous time Markov chain is constructed from a discrete set of exhaustive and mutual exclusive states where changes of state occur randomly.

The mathematical modelling of a system by Markov chains begins with a graph which defines the states of the system, and transition between states is caused by a fault or a repair. Faults can cause stoppage of the system (catastrophic breakdown) or a wearing process; in the latter case the worn states may be considered non-catastrophic faults. The complete mathematical process is described in [90–92], where the final system of equations to be solved is shown in Eq. (19) [93].

19 $$ \left({p}_0,{p}_1,\dots, {p}_{n-1},{p}_n\right)\ast \left(\begin{array}{ccccc}{d}_{00}& {d}_{01}& \cdots & {d}_{0n-1}& 1\\ {}{d}_{10}& {d}_{10}& \dots & {d}_{1n-1}& 1\\ {}\vdots & \vdots & \ddots & \vdots & \vdots \\ {}{d}_{i0}& & {d}_{ij}& & 1\\ {}\vdots & \vdots & \cdots & \vdots & \vdots \\ {}{d}_{n0}& {d}_{n1}& \cdots & {d}_{nn-1}& 1\end{array}\right)=\left(0,0,\dots, 0,1\right) $$

where *p* = (*p*_0_, *p*_1_, …, *p*_*k*_, …, *p*_*m*_) is the vector of probabilities in the stationary state, and the elements *d*_*ij*_ of the matrix are the derivatives of the transition probabilities *p*_*ij*_(*t*) from state *i* to state *j*. Equation (19) may be expressed in matrix form, as shown in Eq. (20).

20 $$ p\ast A=1 $$

Thus, the vector *p* of probabilities in the stationary state can be expressed as in Eq. (21).

21 $$ p=B\ast {A}^{-1} $$

Solving Eq. (21) gives the mean availability *D*_*m*_ of the system analysed. Thus, if the system has *n* − 1 stable operating states, the mean availability is calculated from Eq. (22), where *p*_*i*_ are the coefficients obtained by solving the previous system of equations.

22 $$ {D}_m={p}_0+{p}_1+\dots +{p}_{n-1} $$

Over the life cycle of the systems analysed, the failure and repair rates are constant, and so the exponential distribution of failures and repairs was used. The data used were obtained from the hospital’s Computerised Maintenance Management System. The system of Eqs. (21) was solved using a recursive approach with©Matlab software.

System 1 comprises a hood for preparing cytostatic drugs vertically or by ascending flow. System 2 is used for parenteral feeding and is of the horizontal or lateral ascending flow type. Each system performs different functions which cannot be interchanged. Each system has three section:

1. filter section.
2. gas extraction section.
3. control section with operating alarms.

There is a quarterly preventive check-up, and a more thorough annual check during which the filters and changed and checked, as recommended by the manufacturer. While the preventive maintenance is being carried out, the system is not considered to have failed, as this is programmed work.

A fault at each level means that the system enters a state of breakdown. Each state is observed by the operator who is physically present.

The equivalent failure rate of the systems is calculated from Eq. (23). The equivalent repair rate *μ*_*i*_ for the systems is calculated using Eq. (24). *λ*_*j*_ and *μ*_*j*_ are the failure and repair rates of each of the constituent sections of a system.

23 $$ {\lambda}_i=\sum \limits_{j=1}^k{\lambda}_j $$

24 $$ {\mu}_i=\frac{\lambda_i}{\sum \limits_{j=1}^k\frac{\lambda_j}{\mu_j}} $$

For System 1, λ_11_ and μ_11_ are defined as the failure and repair rates of the filter section. λ_12_ and μ_12_ are the failure and repair rates of the gas extraction section. λ_13_and μ_13_ are the failure and repair rates of the control section with operating alarms.

For System 2, λ_21_ and μ_21_ are defined as the failure and repair rates of the filter section. λ_22_ and μ_22_ are the failure and repair rates of the gas extraction block. λ_23_and μ_23_ are the failure and repair rates of the control section with operating alarms.

Let λ_1_ and μ_1_ be the equivalent failure and repair rates of System 1, and λ_2_ and μ_2_ the equivalent failure and repair rates of System 2.

Particularising for Systems 1 and 2, Eqs. (23) and (24) give:

25 $$ {\lambda}_1={\lambda}_{11}+{\lambda}_{12}+{\lambda}_{13} $$

26 $$ {\mu}_1=\frac{\lambda_1}{\frac{\lambda_{11}}{\mu_{11}}+\frac{\lambda_{12}}{\mu_{12}}+\frac{\lambda_{13}}{\mu_{13}}} $$

27 $$ {\lambda}_2={\lambda}_{21}+{\lambda}_{22}+{\lambda}_{23} $$

28 $$ {\mu}_2=\frac{\lambda_2}{\frac{\lambda_{21}}{\mu_{21}}+\frac{\lambda_{22}}{\mu_{22}}+\frac{\lambda_{23}}{\mu_{23}}} $$

The alternatives considered for optimising the functioning of both these critical pharmaceutical manufacturing systems are the combination of corrective, preventive and predictive maintenance, together with a series of spare parts:

- Corrective and preventive maintenance (CPM). Corrective maintenance is carried out at two levels: the first level is performed by care staff before every use, and the second level by the servicing department of the manufacturer of the system. To this should be added a quarterly preventive check-up, and an annual check-up, also arranged with the manufacturer.
- Corrective and preventive maintenance plus a spare hood (CPM + C). This alternative is defined as being the same as the previous one, but it also considers the availability of a reserve hood, which is included in the plan for corrective and preventive maintenance. The workload of the hoods is shared out, distributing time of use such that each works the same number of annual hours, in each system. It is considered in this case that the failure rate is proportionately lower.
- Corrective and preventive maintenance plus the availability of two spare hoods (CPM + 2C). This alternative is defined as being the same as the previous one, but it also considers the availability of two reserve hoods, which are included in the plan for corrective and preventive maintenance. The workload of the three hoods is shared out proportionally over the year in each system. The failure rate is proportionately lower.
- Corrective, preventive and predictive maintenance (CPPM). This alternative involves the use of a single hood, and corrective maintenance applied by the care staff before each use, and by the technicians of the manufacturer’s servicing department. It also includes a quarterly preventive check-up and an annual one also arranged with the manufacturer’s technical service. To this is added an annual predictive check-up, which monitors hot areas via thermographic analysis, which is outsourced.

The values found for these failure and repair rates in each section, from the record of the time they have been working in the hospital are shown in Table 5. These values are similar for both systems.

**Table 5:** Failure and repair rates for the systems

Failure rate for the filter section (breakdowns/hour)	Failure rate for the gas extraction section (breakdowns/hour)	Failure rate for the control section with operating alarms (breakdowns/hour)	Repair rate for the filter section (repairs/hour)	Repair rate for the gas extraction section (repairs/hour)	Repair rate for the control section with operating alarms (repairs/hour)
*λ*_11_ = 0.000114	*λ*_12_ = 0.000057	*λ*_13_ = 0.000038	*μ*_11_ = 0.0417	*μ*_12_ = 0.021	*μ*_13_ = 0.021

Applying Eqs. (25), (26), (27) and (28) to each optimisation alternative gives the equivalent failure rate *λ*_1_ = *λ*_2_ = 0.000209, and the equivalent repair rate *μ*_1_ = *μ*_2_ = 0.028797.

**Fig. 1:**
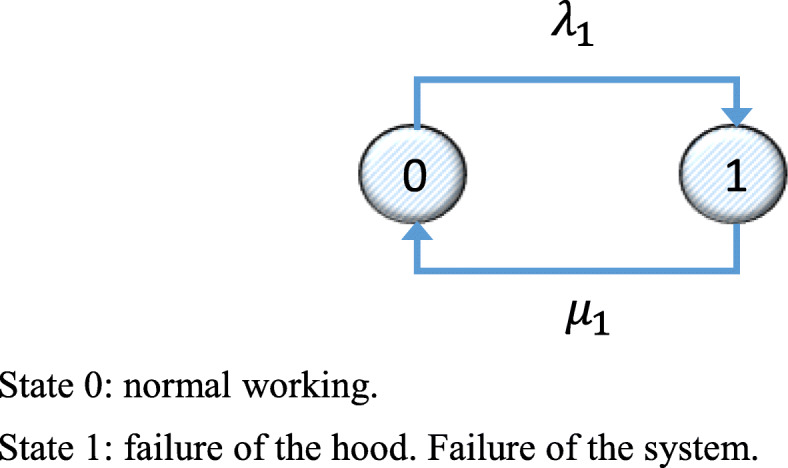
Markov graph for System 1 for the initial installation state. State 0: normal working. State 1: failure of the hood. Failure of the system

The Markov graph considering a single hood is shown in Fig. 1. In this graph, the nodes represent states. A link from State 0 to State 1 describes the probability of a transition from 0 to 1, in this case *λ*_1_. The link from State 1 to State 0 is the probability of a transition between these states, in this case the repair rate *μ*_1_.

Equation (29) shows the transition matrix corresponding to the Markov graph in Fig. 1.

29 $$ \left(\begin{array}{ccc}-{\lambda}_1& {\lambda}_1& 1\\ {}{\mu}_1& -{\lambda}_1-{\mu}_1& 1\\ {}0& {\mu}_1& 1\end{array}\right) $$

Since the subsystem is only operational in State 0, mean availability is *D*_*m*(*CPM*)_ = *p*_0_.

The probability that an element is working is the availability *D*(*t*). According to Creus [94], the general expression for availability is shown in Eq. (30).

30 $$ D(t)=\frac{\mu }{\lambda +\mu }+\frac{\lambda }{\lambda +\mu }\ {e}^{-\left(\lambda +\mu \right)t} $$

Considering infinite time and applying a limit to Eq. (30) give an equation for the mean availability *D*_*m*_.

31 $$ {D}_m=\underset{t\to \infty }{\lim }D(t)=\frac{\mu }{\lambda +\mu } $$

Mean availability when System 1 is functioning normally is thus ${D}_{1m(CPM)}=\frac{\mu_1}{\lambda_1+{\mu}_1}$. Substituting Eqs. (25) and (26) into the previous equation gives that the mean availability in the case of a single hood in System 1 is as shown in Eq. (32). Where k is the number of sections that make up Systems 1 or 2.

32 $$ {D}_{1m(CPM)}=\frac{1}{1+\sum \limits_{j=1}^k\frac{\lambda_j}{\mu_j}}=\frac{1}{1+\frac{\lambda_{11}}{\mu_{11}}+\frac{\lambda_{12}}{\mu_{12}}+\frac{\lambda_{13}}{\mu_{13}}}=0.9928 $$

**Fig. 2:**
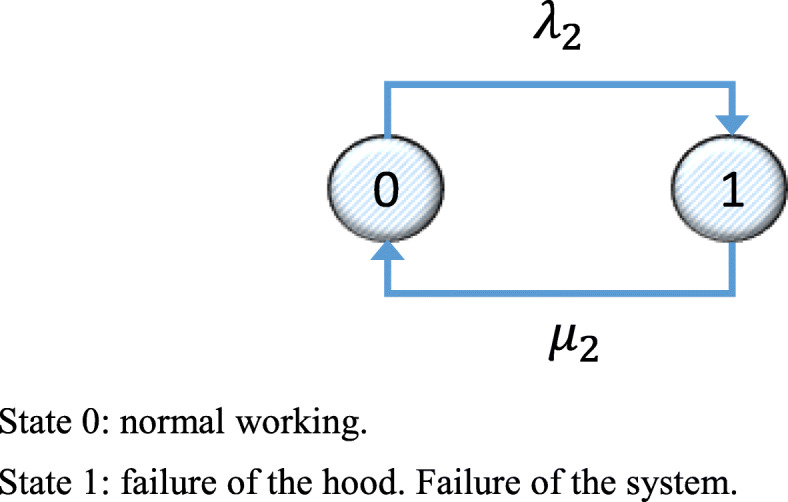
Markov graph for System 2 for the initial installation state. State 0: normal working. State 1: failure of the hood. Failure of the system

Likewise, for System 2, the Markov graph is a s shown in Fig. 2, and the corresponding transition matrix is shown in Eq. (33).

33 $$ \left(\begin{array}{ccc}-{\lambda}_2& {\lambda}_2& 1\\ {}{\mu}_2& -{\lambda}_2-{\mu}_2& 1\\ {}0& {\mu}_2& 1\end{array}\right) $$

The mean availability of System 2, *D*_2*m*(*CPM*)_ is similar to that found for System 1 from Eq. (31), taking into account particular details for its failure and repair rates.

**Fig. 3:**
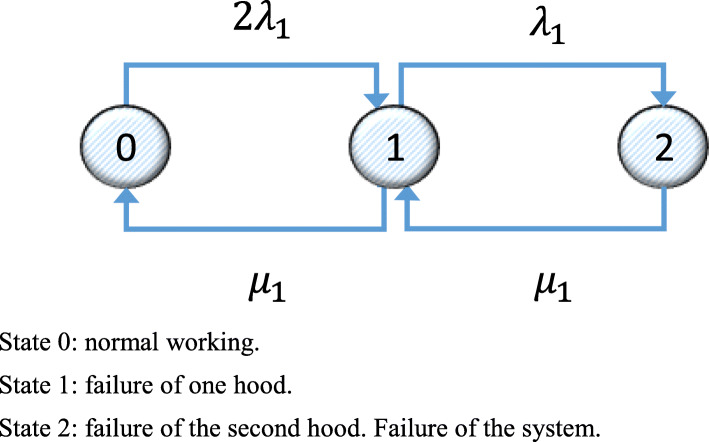
Markov graph for System 1 when there are two hoods. State 0: normal working. State 1: failure of one hood. State 2: failure of the second hood. Failure of the system

Figure 3 shows the Markov graph for System 1 for the alternative with two hoods.

The transition matrix corresponding to the Markov graph in Fig. 3 is shown in Eq. (34).

34 $$ \left(\begin{array}{ccc}-2{\lambda}_1& 2{\lambda}_1& 1\\ {}{\mu}_1& -{\lambda}_1-{\mu}_1& 1\\ {}0& {\mu}_1& 1\end{array}\right) $$

Solving the system of equations in Eq. (21) gives the values of vector *p*. Bearing in mind that System 1 is operative in States 0 and 1, the mean availability of System 1 is *D*_1*m*(*CPM* + *C*)_ = *p*_10_ + *p*_11_ = 0.9999.

**Fig. 4:**
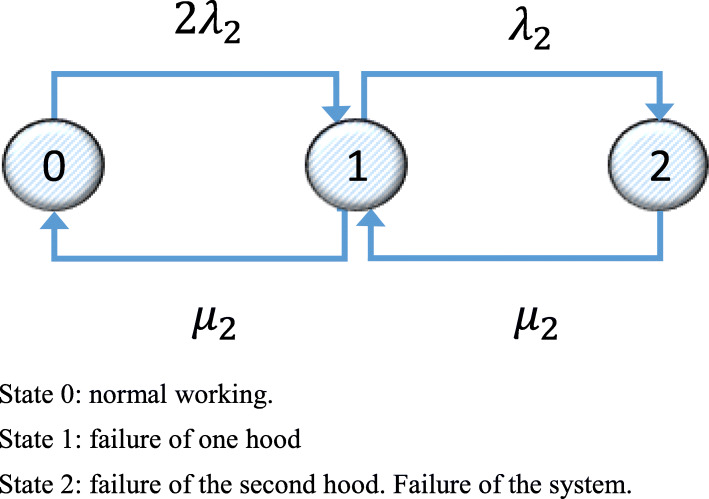
Markov graph for System 2 with two hoods. State 0: normal working. State 1: failure of one hood. State 2: failure of the second hood. Failure of the system

Figure 4 shows the Markov graph for System 2 for the alternative with two hoods.

The transition matrix corresponding to the Markov graph in Fig. 4 is shown in Eq. (35).

35 $$ \left(\begin{array}{ccc}-2{\lambda}_2& 2{\lambda}_2& 1\\ {}{\mu}_2& -{\lambda}_2-{\mu}_2& 1\\ {}0& {\mu}_2& 1\end{array}\right) $$

The results obtained on solving the system of equations in Eq. (21) are similar to those for System 1, as once again System 2 is operative only in States 0 and 1, and the mean availability of System 2 *D*_2*m*(*CPM* + *C*)_ = *p*_20_ + *p*_21_ = 0.9999.

**Fig. 5:**
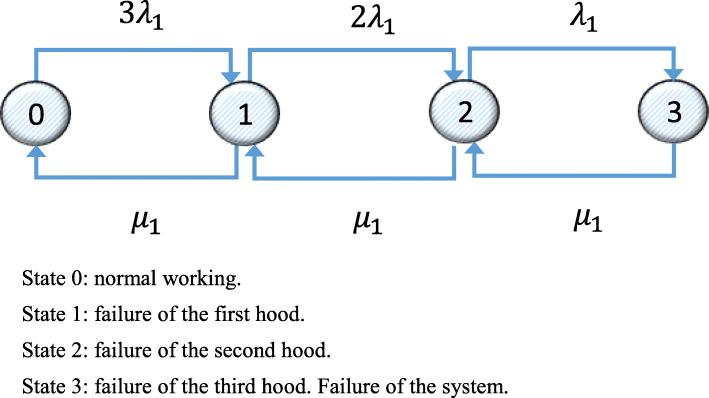
Markov graph for System 1 for three hoods. State 0: normal working. State 1: failure of the first hood. State 2: failure of the second hood. State 3: failure of the third hood. Failure of the system

Figure 5 shows the Markov graph corresponding to System 1 for the alternative with three hoods.

The transition matrix corresponding to the Markov graph in Fig. 5 is shown in Eq. (36).

36 $$ \left(\begin{array}{cccc}-3{\lambda}_1& 3{\lambda}_1& 0& 1\\ {}{\mu}_1& -2{\lambda}_1-{\mu}_1& 2{\lambda}_1& 1\\ {}0& {\mu}_1& -{\lambda}_1-{\mu}_1& 1\\ {}0& 0& {\mu}_1& 1\\ {}0& 0& 0& 1\end{array}\right) $$

Solving the system of equations in Eq. (21) gives values for the probability vector *p* = (*p*_0_, *p*_1_, …, *p*_*m* − 1_, *p*_*m*_) in the steady state, where the mean availability of System 1, *D*_1*m*(*CPM* + 2*C*)_ bearing in mind that the subsystem is operative in States 0, 1 and 2, is *D*_1*m*(*CPM* + 2*C*)_ = *p*_30_ + *p*_31_ + *p*_32_ = 1.

A similar value for mean availability is found for System 2. This is because it has a similar Markov graph, and similar equivalent failure and repair rates, to System 1.

**Fig. 6:**
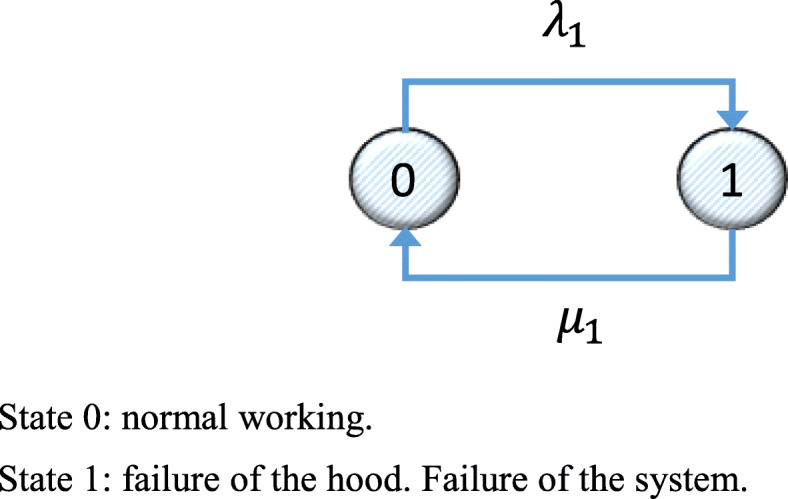
Markov graph for System 1 for the initial installation state. State 0: normal working. State 1: failure of the hood. Failure of the system

The Markov graph for a single hood, applying corrective, preventive and predictive maintenance (CPPM) is shown in Fig. 6. Predictive maintenance would avoid problems in the filter section, and so there could only be failures in the gas extraction section and the control section with working alarms. The equivalent failure rate of the systems is λ_1_ = λ_2_ = 0.000095, and the equivalent repair rate for both systems is μ_1_ = μ_2_ = 0.0210.

The mean availability of System 1, *D*_1*m*(*MCPP*)_ bearing in mind that the system is operative only in State 1, is *D*_1*m*(*CPPM*)_ = *p*_40_ = 0.9954. A similar mean availability is found for System 2.

### Multicriteria model for choice of maintenance policies in preparation systems for cytotoxic drugs

Below is the model, designed with fuzzy TOPSIS, applied to preparation systems for cytotoxic drugs.

**Fig. 7:**
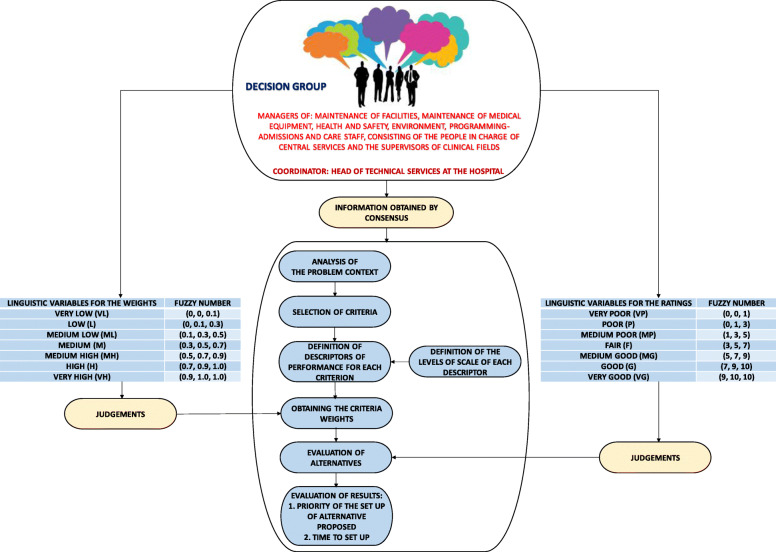
Contributions of the decision group to the model

The multicriteria model was built with the help of a decision group made up of those in charge of different areas of the hospital, such as electro-medicine, facilities maintenance, patient admissions, environment, care staff and health and safety. The decision group was coordinated by the head of Technical Services at the hospital, who is therefore in charge of maintenance, safety and environment at the hospital; he is also greatly experienced in the application of multicriteria techniques. This decision group agreed the decision criteria and their descriptors by consensus. The contributions of the decision group to the model are summarised in Fig. 7.

In order to choose the decision criteria and descriptors, the decision group analysed the literature on the subject.

To avoid ambiguity in the interpretation of the criteria at later stages of the decision process, a descriptor was associated to each criterion or subcriterion to make an operational description. A descriptor is an ordered set of plausible performance levels within a criterion, to describe objectively the impacts of alternatives with respect to that criterion. Scale levels were defined for each descriptor.

The decision group established the following decision criteria and subcriteria:

- Costs. This includes both direct and indirect costs involved with each alternative. The subcriteria of this criterion are investment costs and system maintenance.
  - Investment costs (IC). The mean investment cost per hood, including installation, is €25,000, to be paid off over 10 years. The scale levels of the descriptor Investment cost do not include the cost of annual debt repayment on existing devices already in the system, only those corresponding to the alternatives under consideration. The scale levels of this descriptor, from highest to lowest performance level, are:
    - € 0.
    - € 2000.
    - € 4000
    - € 6000.
  - Maintenance costs (MC). The descriptor Maintenance costs includes the annual maintenance of all the section that make up the system. The scale levels of this descriptor, from highest to lowest performance level, are:
    - € 2000.
    - € 4000.
    - € 6000.
    - € 8000.
- Quality of health care (QH). This measures the impact on care, as a function of mean availability of each alternative. The descriptor associated with this criterion takes into account the time during which the system is operative, via mean availability. This accounts for the existence of stops, breaks in service, or deprogramming, which have consequences for patient care. It should be noted that this criterion only considers the effect of the systems analysed on the quality of care offered by the hospital. Therefore, only technical parameters, such as the mean availability of the systems, are considered. Other things that may affect the quality of care, but which are not related to technical matters in the systems, such as for example those related to the non-availability of care staff to operate the systems, were not analysed. The scale levels of the descriptor, from highest to lowest performance level, are:
  - Mean availability of the system is greater than 0.9990. There are no stoppages or reduction in the healthcare services offered.
  - Mean availability of the system lies between 0.9981 and 0.9990. There is a small stoppage, with no need to interrupt the process.
  - Mean availability of the system lies between 0.9971 and 0.9980. There is a stoppage which requires the attendance of maintenance staff, although the process is not interrupted, the basic functions are maintained and supervised by healthcare staff, until automatic function can be restarted.
  - Mean availability of the system lies between 0.9961 and 0.9970. there is a stoppage, and the service provided by the subsystem must be reduced to below the normal operating level.
  - Mean availability of the system is less than 0.9960. There is a stoppage in the subsystem, and the service offered by the subsystem must be reduced to a level considered critical.
- Impact on hospital management. This criterion measures the impact on the scope of care in the hospital and on the organisation of possible alternatives. It includes the criteria Working environment in the organisation and Impact on scope of health care.
  - Working environment in the organisation (WO). This criterion assesses the existence of differences between care services in the hospital, which may be caused by lack of attention by management to the way resources are assigned. This affects the level of satisfaction of the staff in each care service, but it can also affect the general atmosphere of the hospital. The effect on the regional standing of the hospital was also analysed, as it is reflected in the sense of pride of the workers in the different areas of the hospital. The scale levels of the descriptor Existence of dissatisfaction in areas of health care and effect on hospital standing, from highest to lowest performance level, are:
    - There is no dissatisfaction due to differences in investment of resources in a given area of health care. The hospital increases its standing in the region, and so the staff as a whole feel pride in belonging to it.
    - There are some areas of care where there may be dissatisfaction, on perceiving a reduction in updating and renewal of technology, due to investment of resources in other services or areas. The hospital increases its standing in the region, and so the staff who do not feel aggrieved feel pride in belonging to it.
    - There is no dissatisfaction due to differences in investment of resources in a given area of health care. The hospital neither gains nor loses standing in the region.
    - There are some areas of care where there may be dissatisfaction, on perceiving a reduction in updating and renewal of technology, due to investment of resources in other services or areas, but the standing of the hospital is not affected.
    - This implies a reduction in resources which do exist in other services: space, facilities, equipment or even staff, which leads to a poor working environment in the rest of the hospital.
  - Impact on health care (IH). This assesses the effect on regional health cover, if the service is classed as a reference in the regional health service, and the ability to attend to other areas assigned to the hospital. The scale levels of this descriptor, from highest to lowest performance level, are:
    - It increases health cover in the Regional Health Service. The service provided is classed as a reference within the regional health system, and is able to attend to other areas of the system.
    - It increases health cover in its own catchment area. The service provided increases in the area covered by the hospital, reducing referrals to other areas.
    - It increases health cover at certain times in the catchment area of the hospital. It provides care support, in specific cases, to other areas in the system.
    - There is no extra impact on health cover. It does not provide care support outside its own catchment area, but neither does it require cover from other areas.
    - It requires cover from other hospitals in the regional/national health service. It does not provide any care support outside its catchment area.
- Maintenance planning optimisation (MP). The descriptor associated with this criterion brings together a number of aspects; on the one hand certainty in diagnosing faults or breakdowns in equipment for preparing cytotoxic drugs, and on the other, how far in advance corrective maintenance activities can be programmed, to take into account the impact on provision of care services and optimisation of programming and costs of the maintenance service. The scale levels of this descriptor, from highest to lowest performance level, are:
  - The maintenance technician has reliable diagnostic tools for faults and breakdown, with sufficient advance warning of the failure to programme corrective maintenance without affecting the care service provided. Moreover, corrective actions can be programmed together with those of other hospital systems, which optimises Maintenance Service programming, and maintenance costs.
  - The maintenance technician has reliable diagnostic tools for faults and breakdown, with sufficient advance warning of the failure to programme corrective maintenance without affecting the care service provided. It is not possible to programme maintenance activity together with that of other systems.
  - The maintenance technician has reliable diagnostic tools for faults and breakdown, but with insufficient warning of the failure, requiring corrective maintenance to be carried out immediately. This may lead to programming problems for the Maintenance Service, as it will mean cancelling actions programmed in the short term, which will increase the cost of maintenance, and might affect, to some degree, the operating state of other systems. It is not possible to programme maintenance activity together with that of other systems.
  - The maintenance technician does not have reliable diagnostic tools for all the faults and breakdown analysed, and needs those in more senior positions to take a decision. The advance warning is so short that immediate corrective maintenance action must be undertaken, which may affect the provision of care (by causing the cancellation of programmed services for several hours). This also leads to programming problems for the Maintenance Service, as it means cancelling actions programmed in the short term, which will increase the cost of maintenance, and might affect the operating state of other medical devices. The cost of maintenance is increased. It is not possible to programme maintenance activity together with that of other systems.
  - The maintenance technician does not have reliable diagnostic tools for the faults and breakdown analysed, and needs those in more senior positions to take a decision. There is not enough time to carry out the corrective action before the device stops. This affects the provision of care (by causing the cancellation of programmed services for several days). This also leads to programming problems for the Maintenance Service, as it means cancelling actions programmed in the short term, which can affect the operating state of other medical devices. Maintenance costs are increased. It is not possible to programme maintenance activity together with that of other systems.

The linguistic variables for the weights defined in Table 2 are used to establish the fuzzy weights of the criteria. The results for the fuzzy weightings obtained by the decision group are shown in Table 5. Qualitative judgement intervals were used; for example, in the cost criteria MC and IC, the judgements are low or medium low (L-ML). The resulting fuzzy numbers used cover the range of weights set out in Table 2, corresponding to the linguistically-defined judgement intervals (the results can be seen in the final column of Table 5).

Next, the decision group evaluates each alternative via the linguistically-defined variables from Table 3, obtaining the results of Table 6. It can be seen that for the criterion MC and the alternative CPM + 2C, the decision group considered that the valuation was between fair (3, 5, 7) and medium good (5, 7, 9), and was finally associated with this alternative (3, 6, 9), choosing from the lowest value on the interval to the highest, and the mean as the modal value of the triangular fuzzy number. The case of the criterion IC and the alternative CPM + C is similar. For the criterion MP and the alternatives CPM + C and CPM + 2C the decision group considered that the valuation was between medium good (5, 7, 9) and good (7, 9, 10), and so the final value assigned by the group was (5, 7.5 and 10). The valuation given for the criterion WO and the alternatives CPM + C, CPM + 2C and CPPM was similar.

**Table 6:** Fuzzy weights

Criteria/subcriteria	Linguistic ratings	Fuzzy number weights		
QH	ML	(0.1	0.3	0.5)
MC	L-ML	(0.0	0.3	0.5)
IC	L-ML	(0.0	0.3	0.5)
MP	L	(0.0	0.1	0.3)
IH	L	(0.0	0.1	0.3)
WO	VL	(0.0	0.0	0.1)

The linguistic assessments in Tables 6 and 7 were converted into triangular fuzzy numbers, in order to construct the fuzzy normalised decision matrix in Table 8. This yields the fuzzy weighted normalised decision matrix shown in Table 9.

**Table 7:** Ratings of the decision group for each of the alternatives

Criteria/subcriteria	Alternatives	Linguistic ratings	Fuzzy number ratings
	CPM	VP	(0, 0, 1)
QH	CPM + C	VG	(9, 10, 10)
(maximise)	CPM + 2C	VG	(9, 10, 10)
	CPPM	G	(7, 9, 10)
	CPM	VG	(9, 10, 10)
MC	CPM + C	MG	(5, 7, 9)
(minimise)	CPM + 2C	F-MG	(3, 6, 9)
	CPPM	VP	(0, 0, 1)
	CPM	VG	(9, 10, 10)
IC	CPM + C	F-MG	(3, 6, 9)
(minimise)	CPM + 2C	P	(0, 1, 3)
	CPPM	VG	(9, 10, 10)
	CPM	F	(3, 5, 7)
MP	CPM + C	MG-G	(5, 7.5, 10)
(maximise)	CPM + 2C	MG-G	(5, 7.5, 10)
	CPPM	VG	(9, 10, 10)
	CPM	MP	(1, 3, 5)
IH	CPM + C	F	(3, 5, 7)
(maximise)	CPM + 2C	G-VG	(7, 8.5, 10)
	CPPM	MP	(1, 3, 5)
	CPM	F	(3, 5, 7)
WO	CPM + C	MG-G	(5, 7.5, 10)
(maximise)	CPM + 2C	MG-G	(5, 7.5, 10)
	CPPM	MG-G	(5, 7.5, 10)

**Table 8:** Fuzzy normalised decision matrix

Criteria/subcriteria	Alternatives	Ratings
	CPM	(0.000, 0.000, 0.100)
QH	CPM + C	(0.900, 1.000, 1.000)
(maximise)	CPM + 2C	(0.900, 1.000, 1.000)
	CPPM	(0.700, 0.900, 1.000)
	CPM	(0.900, 1.000, 1.000)
MC	CPM + C	(0.500, 0.700, 0.900)
(minimise)	CPM + 2C	(0.300, 0.600, 0.900)
	CPPM	(0.000, 0.000, 0.100)
	CPM	(0.900, 1.000, 1.000)
IC	CPM + C	(0.300, 0.600, 0.900)
(minimise)	CPM + 2C	(0.000, 0.100, 0.300)
	CPPM	(0.900, 1.000, 1.000)
	CPM	(0.300, 0.500, 0.700)
MP	CPM + C	(0.500, 0.750, 1.000)
(maximise)	CPM + 2C	(0.500, 0.750, 1.000)
	CPPM	(0.900, 1.000, 1.000)
	CPM	(0.100, 0.300, 0.500)
IH	CPM + C	(0.300, 0.500, 0.700)
(maximise)	CPM + 2C	(0.700, 0.850, 1.000)
	CPPM	(0.100, 0.300, 0.500)
	CPM	(0.300, 0.500, 0.700)
WO	CPM + C	(0.500, 0.750, 1.000)
(maximise)	CPM + 2C	(0.500, 0.750, 1.000)
	CPPM	(0.500, 0.750, 1.000)

**Table 9:** The fuzzy weighted normalised decision matrix

Criteria/subcriteria	Alternatives		Ratings	
	CPM	(0.000,	0.000,	0.050)
QH	CPM + C	(0.090,	0.300,	0.500)
(maximise)	CPM + 2C	(0.090,	0.300,	0.500)
	CPPM	(0.070,	0.270,	0.500)
	CPM	(0.000,	0.300,	0.500)
MC	CPM + C	(0.000,	0.210,	0.450)
(minimise)	CPM + 2C	(0.000,	0.180,	0.450)
	CPPM	(0.000,	0.000,	0.050)
	CPM	(0.000,	0.300,	0.500)
IC	CPM + C	(0.000,	0.180,	0.450)
(minimise)	CPM + 2C	(0.000,	0.030,	0.150)
	CPPM	(0.000,	0.300,	0.500)
	CPM	(0.000,	0.050,	0.210)
MP	CPM + C	(0.000,	0.075,	0.300)
(maximise)	CPM + 2C	(0.000,	0.075,	0.300)
	CPPM	(0.000,	0.100,	0.300)
	CPM	(0.000,	0.030,	0.150)
IH	CPM + C	(0.000,	0.050,	0.210)
(maximise)	CPM + 2C	(0.000,	0.085,	0.300)
	CPPM	(0.000,	0.030,	0.150)
	CPM	(0.000,	0.000,	0.070)
WO	CPM + C	(0.000,	0.000,	0.100)
(maximise)	CPM + 2C	(0.000,	0.000,	0.100)
	CPPM	(0.000,	0.000,	0.100)

## Results

The distances ${d}_i^{+}$ and ${d}_i^{-}$ of each weighted alternative from the FPIS and FNIS are calculated using Eqs. (14) and (15), giving the results shown in Table 10. The *CC* are set out in Table 10. The ranking of alternatives is obtained from the value of *CC*, in decreasing order. The best alternative is therefore closest to FPIS = [(1,1,1) (0,0,0) (0,0,0) (1,1,1) (1,1,1) (1,1,1)] and farthest from FNIS = [(0,0,0) (1,1,1) (1,1,1) (0,0,0) (0,0,0) (0,0,0)]. The values (0,0,0) in FPIS arise because the criteria related to costs must be minimised; likewise, in FNIS these values should be (1,1,1).

**Table 10:** The distance measurement and closeness coefficient of each alternative

Alternatives	${d}_i^{+}$	${d}_i^{-}$	***CC***	***Ranking***
CPM	4.494	1.805	0.287	4th
CPM + C	4.059	2.314	0.363	3rd
CPM + 2C	3.824	2.511	0.396	1st
CPPM	3.892	2.404	0.382	2nd

The best result is obtained for Corrective and Preventive Maintenance plus two spare hoods, (CPM + 2C), followed by Corrective, Preventive and Predictive Maintenance (CPPM), Corrective and Predictive Maintenance plus one spare hood (CPM + C), and finally Corrective and Preventive Maintenance (CPM).

**Fig. 8:**
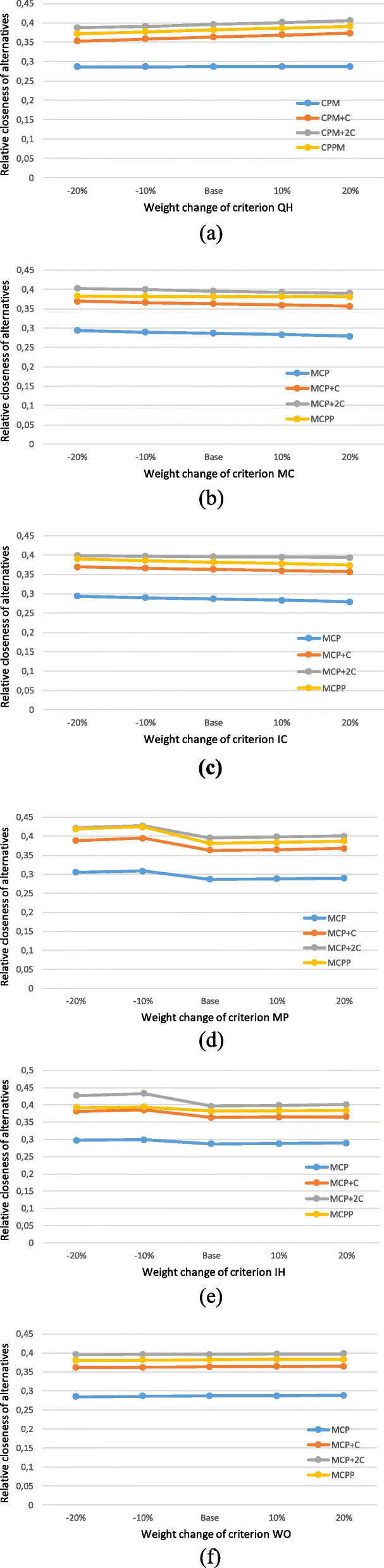
Sensitivity analysis results modifying the weights provided by the group: **A** Quality of Health care (QH); **B** Maintenance Costs(MC); **C** Investment Costs (IC). **D** Maintenance planning optimisation (MP); **E** Impact on care cover (IH); **F** Workplace Environment (WO)

A sensitivity analysis was carried out in order to test the robustness of the model. In this way, the influence of the weight of the criteria on the ranking of alternatives can be analysed. In order to assess the influence of the weight of the criteria on the ranking of alternatives, the aggregated fuzzy weights given by the decision group were modified, increasing and decreasing the weight of each criterion by 10 and 20%. The rankings of the alternatives were evaluated for each of these cases, and the influence of changes in criteria weights on alternatives can be quantified. Figure 8 shows the results obtained by modifying the weights of the decision group.

From the sensitivity analysis, we can see that no modification in the ranking of alternatives is produced by the increase and decrease of the weights provided by the group. Therefore, the model is robust.

## Discussion

The alternative currently applied in the hospital is Corrective and preventive Maintenance (MCP) which means an availability of 0.9928, which can lead to a decrease in services provided to in-patients, as it is sometimes necessary to ask other hospitals for help in preparing medication. This can involve a delay in delivering medication, which can affect the recovery time of patients, and potentially their lives. The multicriteria model here described uses MCP + 2C, which gives an availability of 1; that is, the system is always available. The quality of service is therefore very high, and it can offer services to all the users of the hospital, and also offer cover in the preparation of cytotoxic medicines to other hospitals in the catchment area.

The hospital held that the change of alternative had a value 1 (the highest possible) and also believed that the changes necessary to begin to apply this alternative could be carried out in 1 month. The Maintenance department at the hospital did not have to perform any extra actions over and above those they were already charged with.

The change in costs is €5000 in investment costs, and €2400 annually in increased maintenance costs. However, the improvement in provision of services and quality of care more than make up for the increase in costs.

Unfortunately, just after the end of the study there was a sharp decrease in the hospital budget and changes in the direction of the Technical Services (which includes the maintenance service) that have forced a delay in the implementation of the selected alternative. This was followed by the COVID-19 pandemic, which has made it impossible to make changes in maintenance activities due to oversaturation of the service. This is because the devices are at their maximum level of use, causing more failures and faults. However, it is expected that the selected alternative will be implemented as soon as possible. In addition, it is expected to be able to control, through quantitative indicators, the improvements brought about by the maintenance change introduced, such as a real change in availability, an increase in the number of patients seen per period and a reduction in the waiting time in obtaining treatment for illness or service assistance, as well as a survey on the level of satisfaction of the staff who work with preparation systems for cytostatics.

Intended future lines of research include new alternatives such as Reliability Centred Maintenance or Total Productive Maintenance, as and when the hospital considers its use. It is also useful to review from time to time the judgements of the decision group, as changes in the internal and external environment of the hospital (an increase in maintenance costs, outsourcing of activities, new technologies in medicine production, economic recession, etc.) could change the valuations. The problem can also be analysed using other multicriteria techniques, such as, for example, fuzzy PROMETHEE, fuzzy VIKOR or the fuzzy Best-Worst method, and the results compared. Furthermore, once the alternative proposed by this study is introduced, all the real advantages that accrue, as compared to the alternative previously in place, can be analysed.

## Conclusions

Despite its importance for the patient, care and non-care staff, and the environment, there are no precedents in the literature analysing choices of maintenance policy as applied to systems that prepare cytotoxic drugs. The maintenance of these systems can affect availability of treatment, and thus its effectiveness and the patient’s recovery. It can also cause serious risks to health care staff and the environment should any part of it fail.

This paper sets out, for the first time, a model that contributes to the optimum choice of the combination of maintenance policies and other improvement actions, such as an increase in spare parts or systems, in systems for the personalise preparation of cytotoxic drugs. The model uses fuzzy TOPSIS and a decision group comprising the heads of a number of care and non-care sections, chaired by the assistant manager of Technical Services of the Hospital, who is in charge of maintenance, safety and the environment.

The model, unlike most contributions in the literature, is aimed at actual practice in organisations, and considers as alternatives a combination of different maintenance policies. Also, the alternatives take into account outsourcing of specific maintenance policies or activities, a common practice in many healthcare organisations, and not usually considered in the literature.

The model includes criteria related to the impact on hospital management, such as the effect of one alternative over another on the workplace environment or on care cover. These matters have not been addressed in any previous research, and may serve as a standard for other organisations wishing to use the model for the same purpose.

The model can also be easily applied to other national and international healthcare organisations. This means forming a multi-disciplinary team made up of technical personnel and those responsible for healthcare services, to provide the weightings of the criteria specific to the hospital. These weightings may vary, especially in the case of cost criteria, depending on the public or private nature of the healthcare organisation and the existing budget in each centre. They may even change over time. This may be due, for example, to a greater concern of government policies towards improving the quality of care, providing the centres with higher budgets and making the cost criteria less relevant in the model developed. It would also be necessary to have the average availability of each system that prepares cytotoxic drugs, to be able to evaluate the existing level with respect to the Quality of care (QH) criterion. However, this parameter is usually available in all Hospital Technical Services through a Computerised Maintenance Management System (CMMS). This study includes the criterion Impact on healthcare coverage (HI), which assesses the influence on regional healthcare coverage, whether the service is classified as a reference in the health system of the region and the capacity to care for other areas influence different from that assigned in the hospital. However, other countries may not take into account the influence that a hospital has on an entire region, especially in countries where there is no full public health coverage, as in the case of Spain. However, this criterion could slightly modify its definition in these countries, considering the probability that clients from other areas outside the centre’s coverage radius will move to that healthcare organisation. In addition, for its application to other national and international healthcare organisations, the combination of maintenance policies that are being applied at that time and those that could be applied in the future must be considered as alternatives. In this model, four alternatives are considered: Corrective and preventive maintenance (CPM), Corrective and preventive maintenance plus a reserve hood (CPM + C), Corrective and preventive maintenance plus the installation of two reserve hoods (CPM + 2C) and Corrective, preventive and predictive maintenance (CPPM). Nonetheless, a hospital might not consider it feasible to have a predictive maintenance policy due to the technological requirements and highly specialised training it requires, so it might consider other alternatives instead. However, this would not involve a great effort for the maintenance service, since it would be immediately possible to determine the current alternative that they are applying and the alternatives that would mean different levels of improvement for the hospital.

The results obtained from the model recommend changing the alternative that is currently in use, Corrective and Preventive Maintenance, to Corrective and Preventive Maintenance plus two spare hoods, which would achieve significant improvement in quality of care, and would lower the risks for patients, care and non-care staff, and the environment.

## Data Availability

The datasets used and/or analysed during the current study are available from the corresponding author on reasonable request.

## Abbreviations

WHO: World Health Organization
ISO: International Standard Organization
CM: Corrective Maintenance
PM: Preventive Maintenance
CBM: Condition Based Maintenance
*PdM*: *Predictive Maintenance*
OM: Opportunistic Maintenance
TPM: Total Productive Maintenance
RCM: Reliability Centred Maintenance
TOPSIS: Technique for Order of Preference by Similarity to Ideal Solution
MCDM: Multi-Criteria Decision Making
HTA: Health Technology Assessment
AHP: Analytic Hierarchy Process
SAW: Simple Additive Weighting
ELECTRE: ELimination Et Choix Traduisant la REalité
PROMETHEE: Preference Ranking Organization METHod for Enrichment of Evaluations
MACBETH: Measuring attractiveness through a categorical-based evaluation technique
VIKOR: VIekriterijumsko KOmpromisno Rangiranje
MILP: Mixed Integer Linear Programming
SWARA: Stepwise Weight Assessment Ratio Analysis
WASPAS: Weighted Additive Sum Product Assessment
FAD: Fuzzy Axiomatic Design
ARAS: Additive Ratio Assessment
FS: Fuzzy Set
T2FS: Type-2 Fuzzy Set
TnFS: Type-n Fuzzy Set
AIFS: Atanassov Intuitionistic Fuzzy Set
BVFSL: Bipolar-Valued Fuzzy Set of Lee
BVFSZ: Bipolar-Valued Fuzzy Set of Zhang
CFS: Complex Fuzzy Set
FRS: Fuzzy Rough Set
FSS: Fuzzy Soft Set
GS: Grey Set
HFS: Hesitant Fuzzy Set
THFS: Typical Hesitant Fuzzy Set
IT2FS: Interval Type-2 Fuzzy Set
IVAIFS: Interval-Valued Atanassov Intuitionistic Fuzzy Set
IVFS: Interval-Valued Fuzzy Set
mPVFS: m-Polar-Valued Fuzzy Set
NS: Neutrosophic Set
PFS: Pythagorean Fuzzy Set
SVFS: Set-Valued Fuzzy Set
SS: Shadow Set
VS: Vague Set
TFN: Triangular Fuzzy Number
FPIS: Fuzzy Positive Ideal Solution
FNIS: Fuzzy Negative Ideal Solution
CC: Closeness Coefficient
CPM: Corrective and Preventive Maintenance
CPM + C: Corrective and Preventive Maintenance plus a spare hood
CPM + 2C: Corrective and Preventive Maintenance plus the availability of two spare hoods
CPPM: Corrective, Preventive and Predictive Maintenance
IC: Investment Costs
MC: Maintenance Costs
QH: Quality of Health care
WO: Working environment in the Organisation
IH: Impact on Health care
MP: Maintenance Planning optimisation
CMMS: Computerised Maintenance Management System
